# Detection of the rs10250202 polymorphism in protection of telomeres 1 gene through introducing a new restriction enzyme site for PCR–RFLP assay

**DOI:** 10.1186/s40064-016-2214-5

**Published:** 2016-05-11

**Authors:** Sihua Wang, Xiaoran Duan, Tuanwei Wang, Xiaolei Feng, Pengpeng Wang, Wu Yao, Yongjun Wu, Yiming Wu, Zhen Yan, Feifei Feng, Songcheng Yu, Wei Wang

**Affiliations:** Department of Occupational Health, College of Public Health, Zhengzhou University, Kexue Road 100, Zhengzhou, 450001 Henan Province China; Department of Occupational Health, Henan Institute of Occupational Health, Kangfu Middle Street 3, Zhengzhou, 450052 Henan Province China; Department of Hygiene Toxicology, College of Public Health, Zhengzhou University, Kexue Road 100, Zhengzhou, 450001 Henan Province China; Department of Sanitary Chemistry, College of Public Health, Zhengzhou University, Kexue Road 100, Zhengzhou, 450001 Henan Province China

**Keywords:** Created restriction site, Single nucleotide polymorphism, *Protection of telomeres 1*

## Abstract

Human protection of telomeres 1 (*POT1*) gene is a single stranded telomere binding proteins with a critical role in ensuring chromosome stability. There have been variants of *POT1* gene, and the polymorphisms of *POT1* gene were associated with some diseases. Polymerase chain reaction–restriction fragment length polymorphism (PCR–RFLP) is a traditional method to detect the single nucleotide polymorphism (SNP), and it can be used to detect the polymorphism of rs10250202. But the restriction enzymes required for the detection of the polymorphism of rs10250202 are expensive. So we designed a novel PCR–RFLP assay for genotyping the *POT1* rs10250202 SNP. In the study, a new restriction enzyme cutting site was created by created restriction site PCR (CRS-PCR), and the restriction enzyme *Bcl*I for CRS-PCR was cheaper than other enzymes. After detecting Han Chinese workers, Allele frequencies were found to be 51.54 % for allele *A* and 48.46 % for allele *C* respectively. The PCR results were confirmed by DNA sequencing. CRS-PCR provides a simple, low-cost, practical, and reproducible method.

## Background

Telomere-related genes play an important role in maintaining the integrity of the telomeric structure that protects chromosome ends (Nan et al. 2011). These proteins each bind the G-rich strand of their own telomeric repeat sequence, consistent with a direct role in protecting chromosome ends (Baumann and Cech 2001; Baumann et al. 2002; Hockemeyer et al. 2005). *POT1* is considered to be a significant role for telomere elongation and has an association with telomerase (Izgi et al. 2014). Studies have shown that *POT1*-03 (rs33964002) *A*-allele have a close relationship with breast cancer and can decrease survival rate (Shen et al. 2012); and the polymorphism of *POT1* rs7784168 was significantly associated with lower likelihood of major vessel invasion and overall survival of patients with hepatocellular carcinoma (Jung et al. 2014). Because of the defects of methods in the detecting of rs10250202, *POT1* rs10250202 was little reported in previous study.

PCR–RFLP is a traditional method to detect gene polymorphisms (Mun et al. 2007; Wardak et al. 2005). The advantage of this method is simple, fast and effective, and many restriction enzymes are cheap and commercially available. However, some of the enzymes for a few gene polymorphisms are very expensive, which limited its application. The enzyme for *POT1* gene (rs10250202), *Dpn*I, is the case. Therefore, we created new restriction site by mismatched primers for *POT1* genotypes detection and examined its application in Han Chinese population.

## Methods

### Materials and sequence analysis

The 88 recruiting samples all came from a coke oven plant. The male and female workers are 54 and 34 respectively, and the ages of male and female workers are 22–55 years old and 25–50 years old respectively.

The *POT1* gene information and polymorphism site rs10250202 for *POT1* were obtained from NCBI website (http://www.ncbi.nlm.nih.gov). Sequence analysis shows that the sequence GATC which consist of polymorphism site rs10250202 can be cleaved by *BfuC*I, *Sau3A*I, *Dpn*I, *Dpn*II and *Mbo*I. The cost of above mentioned enzymes is much higher. To find a lower cost PCR–RFLP method, a new *Bcl*I enzyme site was created to detect the *POT1* gene polymorphism.

The prices of above mentioned enzymes from New England BioLabs website (NEB) are as Table 1. From Table 1 we can see that compared to using *Dpn*I enzyme, $70 would be saved when 1000 U enzyme was used to detect 200 samples.

**Table 1:** Identification of the sequence and prices of some kinds of endonucleases in *NEB Co*

Endonucleases	Recognition sequence	$/1000 units
*BfuC*I	GATC	273
*Sau3A*I	GATC	458
*Dpn*I	GATC	101
*Dpn*II	GATC	119
*Mbo*I	GATC	195
*Bcl*I	TGATCA	31

The primers were designed by primer premier 5.0 software. The utilization of the NEB cutter program for restriction enzyme site mapping (http://nc2.neb.com/NEBcutter2/, http://www.neb-china.com) showed that the *Bcl*I restriction enzyme would be useful to distinguish the different alleles of the SNP of rs10250202 in *POT1* gene. Peripheral blood leukocytes of 88 Chinese Han individuals were used as a source of genomic DNA for PCR amplifications by genomic DNA isolation kit (Bio Teke Corporation, Beijing, China). The protocols were approved by Life Sciences of Institutional Review Board of Zhengzhou University Ethics, China (IRB 00006861, FWA00014064); all participants provided written informed consent.

### PCR primers and PCR conditions

The primers designed by primer premier 5.0 software are as follows: the forward primer: 5′-AATTTACTTGATTACGTGTATCTCAAAGC-3′, the reverse primer: 5′-CATATTTCAATATTGTCCAATTAAATGAT-3′.

The PCR was performed in a final volume of 15 μl containing the following: 100 ng of genomic DNA, 0.3 μM each primer(Sangon Biotech, Shanghai, China), 2 × PCR Mix (Lifefeng Biotech, Shanghai, China) and double-distilled water. The cycling conditions are as follows: 95 °C for 5 min; 35 cycles of 95–60–72 °C for 20 s each; and 72 °C for 5 min. Electrophoresis were performed on 2 % agarose gel dyed ethidium bromide for 20 min.

### Genotype of DNA

Enzyme digestion was conducted in a 20 μl final volume consists of 5U of *Bcl*I enzyme (New England Biolab) and 10 μl of PCR products. The reaction was conducted at 50 °C overnight and the digested products were electrophoresed on 3 % agarose gel containing ethidium bromide (0.5 μg/ml) for 40 min, the agarose gel was observed under UV transilluminator.

### PCR products sequencing

PCR products of the sample were Sanger sequenced with Reverse primer by Sangon Biotech Company in Shanghai, China.

### Quality control

We follow in strict accordance with the instructions of DNA extraction kit to extract the genomic DNA. The concentration and purity of DNA sample must meet the PCR requirements. The DNA concentration must be above 50 ng/μl. A260/A280 is in the scope of 1.8–2.0.

PCR lab was divided into four areas, namely, reagent preparation area, sample preparation area, PCR area and product analysis area. The lab must be sterilized by ultraviolet radiation method to avoid cross contamination. Manual Pipettes and PCR instruments must be periodically calibrated to ensure the accuracy.

The positive control and negative control were used in CRS-PCR–RFLP progress. Ten DNA samples were randomly selected to be amplified by PCR and genotyped by enough restriction enzyme digestion. PCR and enzyme-digested products were confirmed by electrophoresis. In PCR progress, one of the above DNA samples of successful PCR was used to be positive control, and water as negative control. The wild and mutant homozygous genotypes of DNA samples were acted as the quality control samples in each enzyme digestion reaction.

## Results

### PCR product analysis

The DNA sequences after PCR according with positions of primers were:

AATTTACTTGATTACGTGTATCTCAAAGCtattttaagattaaagagtaaataagattttggagttgagaccagcattctagtttatgaattctacaatcttgatagagggaaactgtctaga$\underline{{{\text{t}}KATC\boxed{\text{A}}}}$TTTAATT GGACAATATTGAAATATG.

Among the sequence, the capital bases A, T, C and G represent for the same sequences as primers, and *K* represents for T/G (Compatible with polymorphic loci *M* (*A/C*)). The natural base “T” in the DNA sequences was changed into “A” (in frame) after PCR when mismatched base “T” was used in the reverse primer, then a new sequence “t*K*ATCA” (underlined in the above PCR product sequence) was produced for restriction endonuclease *Bcl*I.

### Enzyme cleave identification

**Fig. 1:**
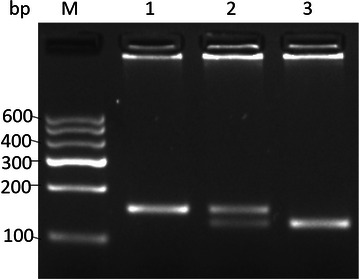
Ethidium bromide stained 3 % agarose gel showing the results of digestion with *BclI*. The 154 bp *POT1* product was digested with *Bcl*I, which does not cut the wild-type Allele *A* sequence but cleaves the mutant-type Allele *C* sequence to yield two restriction fragments of 128 and 26 bp (it’s rarely to be seen in electropherogram). *Lane 1* homozygous wild-type genotype (*POT1*-*AA*); *Lane 2* heterozygous genotype (*POT1*-*AC*); *Lane 3* homozygous mutant-type genotype (*POT1*-*CC*). M: molecular weight marker of 100 bp

According to created restriction site theory, we designed primers to detect the *POT1* rs10250202 polymorphisms. The PCR amplification yielded a product of 154 bp. After digesting with *Bcl*I (digested products separated on 3 % agarose gel were showed in Fig. 1), Apparently, homozygous allele *A* (corresponding to T in the above PCR product sequence) yielded one uncut band (154 bp), the homozygous allele *C* (corresponding to G in the above PCR product sequence) for the mutant type yielded two bands of 128 and 26 bp (it’s rarely to be seen in electropherogram), and the heterozygous type yielded two bands of 154 and 128 bp.

### Genotype analysis

**Fig. 2:**
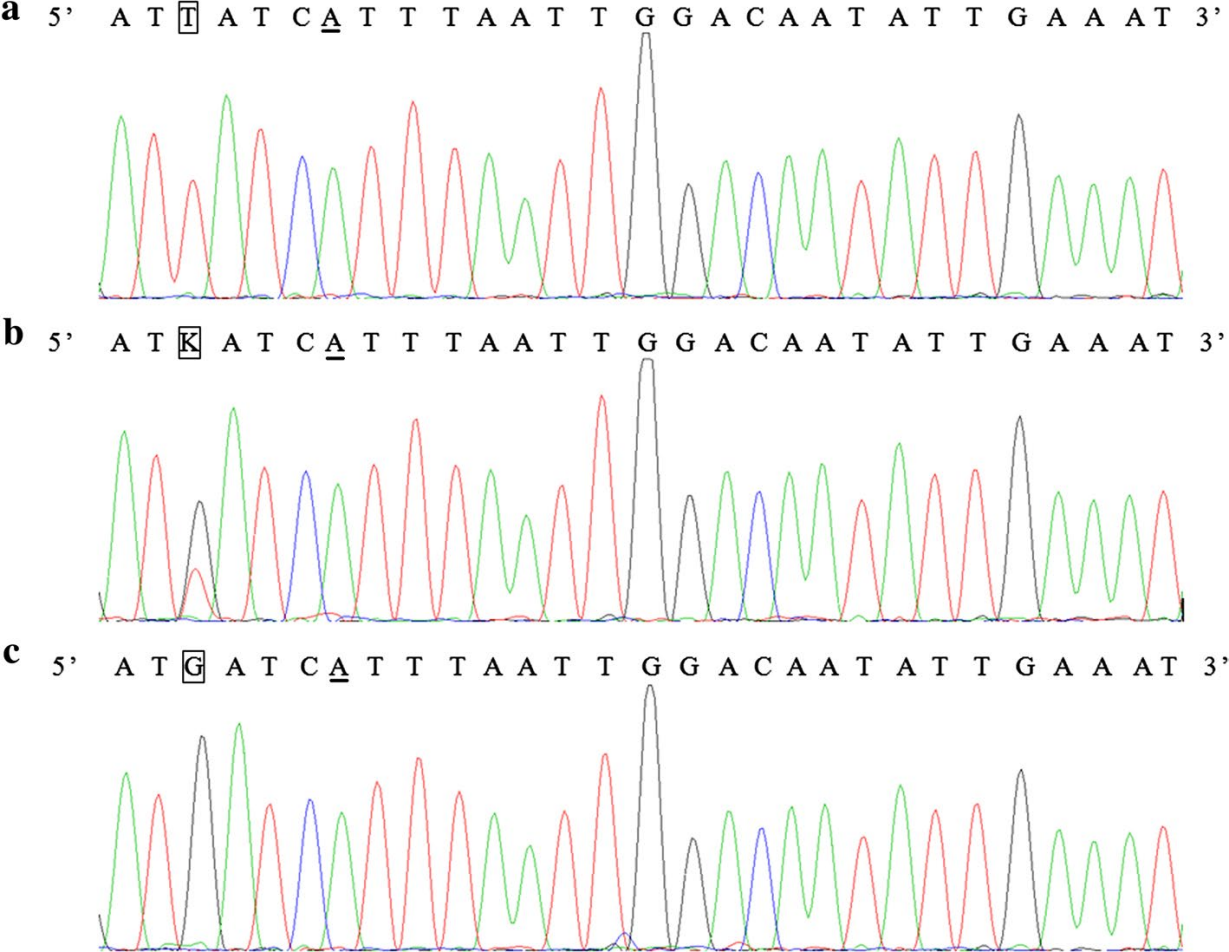
Sequence analysis of the *POT1* gene polymorphism (rs10250202). The underlined base A is mismatched base. The bases in the frames represent the polymorphism sites. The T in frame in **a** represents TT, and corresponds to genotype of AA; the K in frame in **b** represents TG, and corresponds to genotype of AC; the G in frame in **c** represents GG, and corresponds to genotype of CC

Polymorphisms of *POT1* were detected in a coke oven population of 88 Chinese Han individuals. The genotype frequencies were 22 for *AA*, 46 for *AC*, 20 for *CC*, Allele frequencies were found to be 51.54 % for allele *A* and 48.46 % for allele *C* respectively. We also conducted sequencing method which is the golden standard for genotyping to sequence the 88 PCR products, the sequencing data completely matched result from new PCR–RFLP. As shown in the Fig. 2, with the exception of the mismatched base, the sequences of PCR products were consistent with that published in Genebank.

## Discussion

PCR–RFLP method has been used since 1988 (Deng 1988). PCR–RFLP was shown to be a rapid and sensitive method for the detection of gene polymorphism (Fontecha et al. 2015). But this method has some limitations, such as some restriction enzymes *Dpn*I or its isoschizomers are very expensive, which hindered its application, especially in developing countries. In this study, we created new cutting site to detect *POT1* polymorphism of rs10250202, and the matching endoenzyme *Bcl*I was highly cheaper than other enzymes. CRS-PCR–RFLP method also has some limitations. This method cannot detect multiple SNPs in the same reaction, and it can’t realize high-throughput detection. In recent years, there have been about 20 kinds of methods for SNP genotyping. High-throughput methods for SNP genotyping have developed rapidly, for instance, DNA chips/arrays can achieve very high density to accommodate millions of probes on a single chip, and Taqman probe method can type many SNPs on a single slide. These methods can be particularly powerful when an extremely high number of samples are tested. But the above methods’ false positive rate may be much higher when the SNPs are more enough. CRS-PCR–RFLP method can’t realize high-throughput detection, but its accuracy is relatively high. In this article, this new method got the same results as sequencing method. This method is fit for small samples’ study, and it is reliable, economic and low-cost.

In conclusion, here we report a simple and economical technique for analysis of *POT1* polymorphism of rs10250202 site which can be used widely in the study of the genetics of local research.

## Abbreviations

*POT1*: human protection of telomeres 1
PCR–RFLP: polymerase chain reaction–restriction fragment length polymorphism
SNP: single nucleotide polymorphism
CRS: created restriction site
